# R-loops in neuronal aging

**DOI:** 10.18632/aging.205070

**Published:** 2023-09-13

**Authors:** Hana Hall

**Affiliations:** 1 Biochemistry Department, Purdue Institute for Integrative Neuroscience, Purdue University, West Lafayette, IN 47906, USA

**Keywords:** R-loops, neuron, transcription, genome instability, neurodegeneration

As organisms age, they experience time-related decline in their physiological function, which is accompanied by increased incidence of morbidity and mortality. Aging is proposed to be driven by twelve pathways, or hallmarks, such as genome instability, epigenetic alterations, loss of proteostasis, and chronic inflammation [1]. Importantly, modulations of these aging pathways may provide a therapeutic opportunity to slow or reverse the aging process. We and others have shown that aging is also characterized by changes in gene expression, which leads to dysregulation of multiple biological processes associated with aging. While some gene expression changes can be explained by environmental factors, intracellular regulation, or loss of cellular identity, it has been shown that a process of transcription itself can contribute to gene expression dysregulation.

During transcription, a nascent RNA commonly anneals back to the template DNA strand, thus generating an RNA-DNA hybrid and displaced ssDNA, also called an R-loop [2]. R-loops have been shown and studied in a wide range of organisms and while they have important regulatory roles, persistent R-loops can be detrimental to cell function and survival, having been closely linked to both gene expression dysregulation and increased genome instability. Therefore, it is not surprising that an imbalance in R-loop homeostasis is linked to multiple human pathologies, including cancer and neurological and autoimmune diseases. To mitigate the consequences of persistent R-loops, cells possess mechanisms to resolve them and prevent their formation. For example, R-loops can be resolved by dedicated enzymes, such as the RNA/DNA helicase Senataxin, and the RNA-DNA specific ribonuclease H (RNase H). In addition, topoisomerases suppress R-loop formation by resolving negative DNA supercoiling, and various RNA binding, processing, and export proteins sequester nascent transcripts from the template, or help export them from the nucleus. Since the first *in vivo* demonstrations of R-loops in the 1990s, and especially during the last decade, R-loops have been primarily studied in dividing cells, with very limited exploration in post-mitotic cells such as neurons. Nevertheless, due to their high level of transcription and post-transcriptional RNA processing, neurons might be particularly vulnerable to perturbations in gene expression due to increased R-loop formation.

Our transcriptome and protein profiling of aging *Drosophila* *melanogaster* photoreceptor (PR) neurons and eyes, respectively, revealed decreased levels of several factors involved in RNA metabolism [3, 4]. This prompted us to hypothesize that R-loop levels increase during aging. In our recent study, we demonstrated that R-loops accumulate in fly PR neurons by middle age and significantly increase into late-life stages [5]. To further explore the connection between R-loops and aspects of gene expression such as transcription level, gene sequence, and DNA topology during aging, we next sought to characterize R-loop distribution genome-wide. We combined our nuclear pull-down technique with the recently developed Map-R, which uses catalytically inactive RNase H1 tethered with micrococcal nuclease to produce RNA-DNA hybrid fragments [6], and adapted it to cell-type specific R-loop mapping. We demonstrate that genomic R-loop coverage significantly increases with age, and their age-related distribution is associated with specific features such as transcript levels and GC content.

Further analysis revealed that long genes are particularly susceptible to R-loop formation in aging neurons, possibly through an increase in topological stress generated during transcription elongation. During transcription, movement of elongating RNA polymerase creates underwinding of DNA behind the polymerase, where the more relaxed DNA is prone to RNA transcript invasion and R-loop formation. To resolve torsional stress that occurs during transcription, cells use topoisomerase enzymes to solve both RNA and DNA topological problems. Topoisomerase 3 β (Top3β) is a highly conserved, dual-activity RNA/DNA topoisomerase whose loss of function has been associated with neurological disorders in humans and a decreased lifespan in mice. Given that we observed decreased Top3β protein levels in eyes of aging flies, we decided to test its role in gene expression during neuronal aging. Similar to previous studies in mammalian cells, we demonstrated that Top3β plays a role in R-loop biology during aging in *Drosophila*. We observed approximately a 10% increase in R-loop levels in flies depleted for Top3β, accompanied by decreased visual response, and decreased expression of a subset of long genes. Importantly, reduction in R-loop levels by eye-specific over-expression of either Top3β or RNase H1 enzyme mitigated age-associated loss of visual function in flies, highlighting the importance of maintaining R-loop homeostasis in gene expression during aging.

Our data is in line with a recent report by Gyenis et al., showing substantial mammalian RNA polymerase (RNAP) II stalling during transcription elongation, which leads to a gene-length dependent decrease in transcription [7]. The authors propose that this age-related transcriptional stress is increased by spontaneous DNA damage, which affects long genes at a higher rate. Since one of the consequences of persistent R-loops is inhibition of elongating RNAP, accumulation of R-loops during aging may also be a significant contributor to transcriptional stress. Our R-loop profiling of aging PR neurons did not identify any *de novo* R-loops. Instead, we observed widening of R-loop peaks, which suggests that their increased levels result from stabilization or expansion. Therefore, we propose that age-associated R-loop accumulation is not due to increased formation, but rather to failure to resolve existing R-loops. Notably, recent evidence shows that stable R-loops can be processed into DNA breaks, followed by export of cleaved RNA-DNA hybrids into the cytoplasm, where they can trigger the innate immune response [8]. This points to a pathological role of RNA-DNA hybrids outside of the nucleus.

Our study provides first evidence of R-loop accumulation in aging neurons and a contributing role in loss of neuronal function during aging. Undoubtedly, R-loops are at the crossroads of several hallmarks of aging, namely transcriptional stress, genome instability, and chronic immune response. Targeting R-loop levels thus may help restore these pathways to a normal/healthy state and slow down or prevent the onset of age-dependent neurodegenerative diseases.
